# Antimicrobial Activity of *Lactococcus lactis* subsp. *lactis* Isolated from a Stranded Cuvier’s Beaked Whale (*Ziphius cavirostris*) against Gram-Positive and -Negative Bacteria

**DOI:** 10.3390/microorganisms9020243

**Published:** 2021-01-25

**Authors:** Akihiko Suzuki, Miwa Suzuki

**Affiliations:** Laboratory of Aquatic Animal Physiology, Department of Marine Science and Resources, Graduate School of Bioresource Sciences, Nihon University, 1866 Kameino, Fujisawa 252-0880, Japan; brak19502@g.nihon-u.ac.jp

**Keywords:** bacteriocin, fecal bacteria, lactic acid bacteria, *Lactococcus lactis*, marine mammal, whale

## Abstract

In the present study, we isolated and characterized *Lactococcus lactis* (*L*. *lactis*) subsp. *lactis* from a female Cuvier’s beaked whale (*Ziphius cavirostris*) stranded in Shizuoka, Japan. Only five isolates (CBW1-5), grown on Lactobacilli de Man Rogosa Sharpe (MRS) agar plates prepared using 50% artificial seawater, were positive in *L*. *lactis* species-specific primer PCR. Their 16S rRNA sequences were highly similar to those of *L*. *lactis* subsp. *lactis* JCM 5805^T^. The Gram reaction, motility, gas production from glucose, catalase production, and growth conditions were consistent with those of the type strain. Additionally, carbohydrate utilization of the strains was consistent with previously reported marine organism-derived strains. The pH-neutralized cell-free culture supernatant of strain CBW2 inhibited the growth of *Bacillus subtilis* subsp. *subtilis* ATCC 6051 and *Vibrio alginolyticus* ATCC 17749, whereas protease treatment eliminated or diminished its inhibitory activity. The strain possesses a precursor of the nisin structural gene (*nisA*), which showed 100% homology with nisin Z, and nisin biosynthesis-related genes (*nisB*, *nisC*, *nisT*, *nisP*, *nisF*, *nisI*, and *nisRK*), suggesting that the strain produces a nisin-like substance. This study provides fundamental information on whale-derived *L*. *lactis* subsp. *lactis* which may be useful for reducing the carriage of *B*. *subtilis* subsp. *subtilis* and *V*. *alginolyticus*.

## 1. Introduction

Some strains of lactic acid bacteria (LAB), such as *Enterococcus*, *Lactobacillus*, *Lactococcus*, and *Pediococcus*, have received attention as beneficial organisms [1]. LAB create a low-pH environment, by fermenting several nutrients, and produce antimicrobial factors, which can prevent contamination by pathogenic bacteria [1,2]. In addition, the interaction between LAB and the intestinal mucosa of the host is associated with immunostimulating and immunomodulatory effects [3]. Therefore, many studies have reported the potential benefits of using various LAB strains in the food industry [4,5] and LAB-associated health improvement in animals and humans [6,7,8].

Bacteriocins are gene-encoded, ribosomally-synthesized antimicrobial peptides produced by both Gram-positive and -negative microorganisms [9]. Bacteriocins derived from LAB have a wide variety of potential applications, such as preventing contamination by pathogenic bacteria in food and as alternatives to antibiotics for medical and veterinary use [10]. Nisin, produced by some strains of *L*. *lactis*, is a widely studied bacteriocin. This heat-stable, non-toxic, cationic lantibiotic belongs to class I bacteriocins, according to the classification criteria of Klaenhammer [9]. It consists of 34 amino acids, including several unusual amino acids such as dehydroalanine, dehydrobutyrine, aminobutyric acid, lanthionine, and *β*-methyllanthionine [9,11]. Nisin can kill closely related bacteria but also a wide range of Gram-positive bacteria, including *Staphylococcus aureus* (*S*. *aureus*), *Bacillus* spp., and *Clostridium* spp., by forming pores in the cell membrane via binding to lipid II, a bacterial cell wall precursor, and causing the subsequent efflux of small cytoplasmic contents such as amino acids, nucleotides, and ions [11,12]. However, it cannot kill Gram-negative bacteria because of the structure of the outer membrane barrier, outward cytoplasmic membrane, and peptidoglycan layer, which is composed of lipopolysaccharide molecules in the outer leaflet and glycerophospholipids in the inner leaflet [11,13]. Because of its potency against various Gram-positive bacteria, nisin is widely used as a food preservative in processed cheeses, dairy products, and canned foods [9,14].

Few LAB strains have been isolated from cetaceans. *Lactobacillus salivarius* (*Lact. salivarius*) was isolated from the rectal contents of a captive common bottlenose dolphin (*Tursiops truncatus*). Its strains inhibit the growth of pathogens in marine mammals and humans and stimulate the production of tumor necrosis factor in mammalian cells [15]. *Lactobacillus ceti* (*Lact. ceti*) 142-2^T^ was isolated from the liver and lungs of a stranded Cuvier’s beaked whale (*Ziphius cavirostris*) [16], and *Weissella ceti* (*W*. *ceti*) 1119-1A-09^T^ was isolated from several organs of beaked whales (*Mesoplodon bidens*) [17]. *Lactococcus garvieae* (*L. garvieae*), which is suspected to be responsible for diseases in fish, was isolated from the muscle and kidney of a stranded common bottlenose dolphin [18,19]. *L*. *lactis* is not normally considered as part of the commensal microbiota [10] but has been isolated not only from terrestrial mammals [20] but also from marine organisms [21,22,23,24]. In addition, various studies showed that several *L*. *lactis* strains can survive transit through the mammalian gastrointestinal tract [10,25]. Therefore, *L*. *lactis* strains may also exist in cetacean gastrointestines as indigenous microbial members. In one study, *L*. *lactis* strains were isolated from cetaceans, specifically only from stranded harbor porpoises (*Phocoena phocoena*), and the clinical relevance between the isolated *L*. *lactis* strains and pneumonia as a secondary infection was stated, however, the fundamental characteristics of the strains were not investigated [26]. Thus, our knowledge regarding *L*. *lactis* strains in cetacean organs is limited. In this study, we aimed to determine the phenotypic characteristics and antimicrobial activities of *L*. *lactis* subsp. *lactis* strains isolated from a fecal sample of a stranded Cuvier’s beaked whale during an investigation of the gastrointestinal microbiota of the whale.

## 2. Materials and Methods

### 2.1. Sample Collection and Stranded Whale Species Identification

A female stranded whale with a body length of 5.61 m was found on the Miho coast, Shimizu, Shizuoka, Japan (35°00′38.6″ N, 138°31′53.2″ E, Figure S1) on 18 September 2019. After wiping the skin around the vent using 70% ethanol, a fecal sample was collected using a sterile spoon and stored in Kenki-Porter II (Terumo, Tokyo, Japan), an anaerobic transport vial tube, at 4 °C until further experimentation in the laboratory.

The species of the stranded whale was identified by morphological examination and genetic investigation using a primer set (Table 1) for amplification of a 550-bp fragment of the mitochondrial DNA D-loop region. The reaction mixture consisted of 0.5 μL of each primer (10 μM), 2.5 μL of 10× PCR buffer for Blend Taq (Toyobo, Osaka, Japan), 2.5 μL dNTPs (2 mM), 0.25 μL of Blend Taq Plus polymerase (Toyobo), 17.75 μL of distilled water, and 1 μL of template crude DNA. The PCR conditions were as follows: initial denaturation at 94 °C for 2 min, 30 cycles of denaturation at 94 °C for 30 s, annealing at 55 °C for 30 s, and extension at 72 °C for 30 s.

### 2.2. LAB Isolation from the Fecal Sample

A loopful of fecal sample was inoculated on Lactobacilli MRS agar plates (BD Difco, Franklin Lakes, NJ, USA), prepared with 50% artificial seawater and containing 1% CaCO_3_, and incubated at 37 °C until colonies developed under anaerobic conditions, established using Anaeropack (Mitsubishi Gas Chemical Company, Tokyo, Japan) and an anaerobic jar (Mitsubishi Gas Chemical Company). The screening of bacterial acid production was completed by checking for the formation of a clear zone around the colony on agar plates. The developed colonies showing acid production were stored as 20% glycerol stocks at −80 °C for subsequent phylogenic and biochemical analyses.

### 2.3. Screening for L. lactis Using Species-Specific Primers and 16S rRNA Gene Sequencing

Bacterial DNA was extracted from the bacterial strains by bead disruption in sterile 10% Triton X-100 (MP Biomedicals, Irvine, CA, USA) and then used as a template for PCR amplification. As an initial screening for *L*. *lactis*, PCR amplification was conducted as previously described [22] using a universal forward primer and an *L. lactis*-specific reverse primer (Table 1). The PCR amplicons were subjected to electrophoresis on a 2% agarose gel, and the presence of expected bands was examined using a Gel Doc EZ Imager (Bio-Rad, Hercules, CA, USA) under UV light.

Approximately 1500 bp of the bacterial 16S rRNA gene region were amplified by the universal primers (Table 1). The PCR conditions were as follows: initial denaturation at 94 °C for 2 min, 25 cycles of denaturation at 94 °C for 30 s, annealing at 55 °C for 30 s, and extension at 72 °C for 90 s. The composition of the PCR mixture was the same as that described above. The PCR amplicons were purified using a Wizard SV Gel and a PCR clean-up system (Promega, Madison, WI, USA) and directly sequenced using an ABI 3130xl Genetic Analyzer (Applied Biosystems, Foster City, CA, USA) with a BigDye™ Terminator v3.1 Cycle Sequencing Kit (Applied Biosystems). The obtained sequences were subjected to a similarity-based search against quality-controlled databases of 16S rRNA sequences in EzBioCloud [32]. Multiple sequence alignments with the related species in the genus *Lactococcus* obtained from the NCBI GenBank database (https://www.ncbi.nlm.nih.gov/) were performed using the CLUSTAL W program [33]. A phylogenetic tree was reconstructed using the neighbor-joining method [34] and Kimura’s two-parameter model [35] in MEGA X ver. 10.1.8 [36], and the tree topology was evaluated using 1000 bootstrap replicates.

### 2.4. Classification of L. lactis Subspecies Using PCR-Restriction Fragment Length Polymorphism Analysis

Five isolates showing positive results by PCR amplification with *L*. *lactis* species-specific primers and the highest similarity to the 16S rRNA gene sequences of *L*. *lactis* subspecies strains were investigated using a primer set (Table 1) to amplify the *gadB* gene encoding glutamate decarboxylase, which was observed in *L*. *lactis* subsp. *lactis* but not in *L*. *lactis* subsp. *cremoris* [37]. The PCR conditions were as follows: initial denaturation at 94 °C for 2 min, 35 cycles of denaturation at 94 °C for 30 s, annealing at 50 °C for 30 s, extension at 72 °C for 1 min, and final extension at 72 °C for 5 min. Subsequently, the amplified fragments were digested using AseI endonuclease (Nippon Gene, Tokyo, Japan) following the manufacturer’s instructions. The expected PCR amplicon sizes were approximately 600 and 560 bp for *L*. *lactis* subsp. *lactis* and subsp. *cremoris*, respectively. The amplicon of the *L*. *lactis* subsp. *lactis* strain was cut into two fragments (approximately 190 and 410 bp in length) by the enzyme, whereas that of the *L*. *lactis* subsp. *cremoris* strain was not [31]. The cleaved amplicons were visualized on a 2% agarose gel using a Gel Doc EZ Imager (Bio-Rad).

### 2.5. Random Amplified Polymorphic DNA (RAPD)-PCR Analysis

Genomic DNA was extracted from the bacterial strains using a NucleoSpin Microbial DNA kit (Macherey-Nagel, Düren, Germany) according to the manufacturer’s instructions. To analyze the genetic diversity of the five isolates, random amplified polymorphic DNA (RAPD)-PCR analysis using M13 primer (5′-GAGGGTGGCGGTTCT-3′, [38]) was performed. The reaction mixture consisted of 2 μL of each primer (50 μM), 5 μL of 10× PCR buffer for Blend Taq (Toyobo), 5 μL of dNTPs (2 mM), 0.5 μL of Blend Taq Plus polymerase (Toyobo), 36.5 μL of distilled water, and 1 μL of template DNA (30 ng μL^−1^). The PCR conditions were as follows: initial denaturation at 94 °C for 2 min, 35 cycles of denaturation at 94 °C for 30 s, annealing at 50 °C for 1 min, and extension at 72 °C for 2 min. The PCR amplicons were visualized on a 1.5% agarose gel using a Gel Doc EZ Imager (Bio-Rad).

### 2.6. Biochemical Characterization

All strains were subjected to Gram reaction and motility tests, and the production of gas from glucose and catalase was assessed. The Gram reaction test were performed using 3% potassium hydroxide [39]. For the motility test, a single colony of the strains was inoculated into lysine indole motility semisolid agar (Eiken Chemical, Tokyo, Japan), and the presence or absence of the diffusion of bacteria growth was checked after incubation at 37 °C for 24 h. Gas production from glucose was tested using agar containing 1% glucose, 1.5% peptone, 0.5% sodium chloride, and 0.002% phenol red in Durham tubes [22]. Catalase production was examined in the presence of gas bubbles within 30 s after adding the purified strains to 1 mL of hydrogen peroxide (Fujifilm Wako Pure Chemical Corporation, Osaka, Japan). The final pH of the Lactobacilli MRS broth after two days of incubation at 37 °C was measured using an F-50 pH meter (Horiba, Kyoto, Japan). The carbohydrate fermentation profile was tested using the API 50 CH system (bioMérieux, Marcy-l’Étoile, France) with API 50 CHL medium (bioMérieux) following the manufacturer’s instructions.

To classify the *L*. *lactis* subspecies, the strains were incubated for two days in Lactobacilli MRS broth at pH 4.0, 9.2, and 10.0 at 37 °C, and on Lactobacilli MRS agar plates at 10 °C, 40 °C, and 45 °C. Additionally, the sodium chloride tolerance of the strains was investigated by incubation on Lactobacilli MRS agar plates containing 1%, 2%, 3%, 4%, 5%, 6%, 7%, 8%, 9%, 10%, and 15% sodium chloride at 37 °C for two days.

### 2.7. Antimicrobial Activity Assay

The agar well diffusion assay was performed as described previously [6,40], with some modifications. The suspension of the Gram-positive and -negative indicator bacterial strains (Table 2) was adjusted to McFarland Standard No. 0.5 and inoculated onto Muller Hinton agar plates (BD Difco). Well holes (5 mm in diameter) were hollowed out using a sterile alloy pipe and filled with 100 µL of cell-free supernatant of Lactobacilli MRS broth of each *L*. *lactis* subsp. *lactis* strain (adjusted to pH 7.0 by 2 N NaOH) obtained by repeated centrifugation at 5000× *g* for 10 min and 0.22-μm filter sterilization. The Muller Hinton agar plates with each indicator strain were incubated under the conditions shown in Table S1. The pH-neutralized, cell-free supernatants from the strains were treated with 3 mg mL^−1^ (final concentration) proteinase K (Fujifilm Wako Pure Chemical Corporation), trypsin (Fujifilm Wako Pure Chemical Corporation), lysozyme (MP Biomedicals), catalase (Tokyo Chemical Industry, Tokyo, Japan), pepsin (Nacalai Tesque, Kyoto, Japan), and lipase (Nacalai esque). The agar well diffusion assay of the enzyme-treated cell-free supernatants was performed against the indicator bacterial strains, whose growth was inhibited by the pH-neutralized, cell-free supernatants. Antimicrobial activity was determined by measuring the diameter (mm) of the inhibition zone around the well. Strains with inhibition zones less than 6 mm in diameter, between 6 and 10 mm, and more than 10 mm were classified as non-inhibitors, intermediate inhibitors, and strong inhibitors, respectively. The assay was performed in triplicate, and the inhibition zone of enzyme-treated, cell-free supernatants was compared with that of the pH-neutralized, cell-free supernatant treated as a control, by one-way analysis of variance followed by Dunnett’s test.

### 2.8. Genetical Analysis for Bacteriocin Production Potential

All *L*. *lactis* subsp. *lactis* strains were screened for the presence of the precursor of the nisin structural gene (*nisA*), which encodes the prepeptides of nisin, lacticin 481 and 3147, and lactococcin A, B, M, and 972, using the primer sets shown in Table 3. PCR amplification was performed under the following conditions: initial denaturation at 94 °C for 2 min, 35 cycles of denaturation at 94 °C for 30 s, annealing at 45 °C (for nisin) or 50 °C (for lacticin 481 and 3147 and lactococcin A, B, M, and 972) for 30 s, extension at 72 °C for 30 s, and final extension at 72 °C for 5 min.

Subsequently, all strains were subjected to PCR amplification targeting nisin biosynthesis-related genes. The primer sequences and targeted genes for each assay are shown in Table 3. The PCR conditions were as follows: initial denaturation at 94 °C for 2 min, 35 cycles of denaturation at 94 °C for 30 s, annealing at 42 °C (for *nisC*), 45 °C (for *nisP* and *nisRK*), and 55 °C (for *nisB*, *nisT*, *nisI*, and *nisF*) for 30 s, extension at 72 °C for 1 min, and final extension at 72 °C for 5 min.

The resulting amplicons for *nisA* were purified and sequenced as described above. The obtained DNA sequences were translated into amino acid sequences using the ExPASy translate tool [41], and then the blastp-based protein sequence similarity search tool in RiPPMiner, a ribosomally synthesized and post-translationally modified peptide (RiPP) database [42], was used to find the closest neighbors of RiPP peptides and predict the RiPP class of the gene product.

## 3. Results

### 3.1. Isolation of L. lactis Strains from a Stranded Cuvier’s Beaked Whale

The morphology of the stranded whale was identical to that of Cuvier’s beaked whale, and the mitochondrial DNA D-loop region sequence of the whale was homologous to that of the species (GenBank accession number: AB610404) at 100% identity.

**Figure 1:**
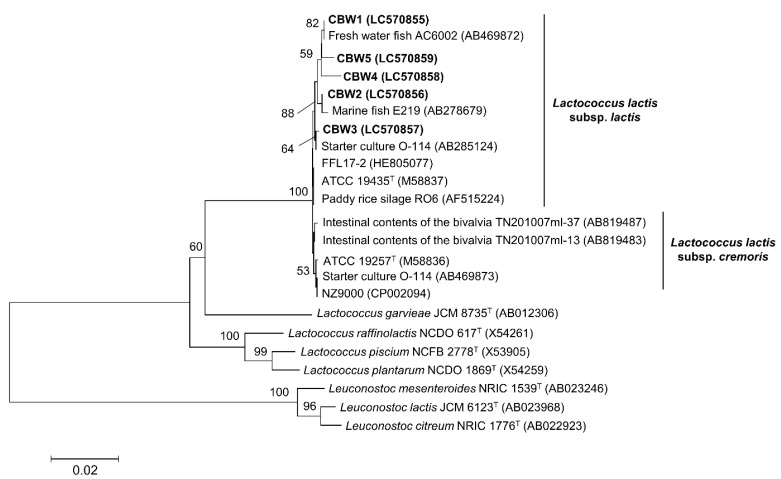
Phylogenetic tree generated using the neighbor-joining method and Kimura’s two-parameter model based on the 16S rRNA gene sequences (1326 bp) representing the relationships between the isolated strains CBW1–5 and related species in the genus *Lactococcus*. The accession numbers are shown inside the parentheses. Only >50% bootstrap values based on 1000 replicates are shown. The scale bar represents the evolutionary distance of the nucleotide substitutions per site. Some species in the genus *Leuconostoc* are used as an outgroup.

We isolated 46 potential LAB isolates based on acid production. Of these, five strains, namely CBW1–5, showed *L*. *lactis*-specific bands (Figure S2). According to 16S rRNA gene sequencing, five strains had sequences highly homologous to that of *L*. *lactis* subsp. *lactis* JCM 5805^T^ (99.04–99.93% similarity) (Table 4). These strains were clustered in the same group as *L*. *lactis* subsp. *lactis* using the neighbor-joining method and Kimura’s two-parameter model, with 100% bootstrap values based on 1000 replicates (Figure 1). In addition, the band patterns of the five strains amplified by RAPD-PCR analysis using the M13 primer differed from each other (Figure S3).

### 3.2. Classification of L. lactis Subspecies in the CBW1–5 Strains

**Figure 2:**
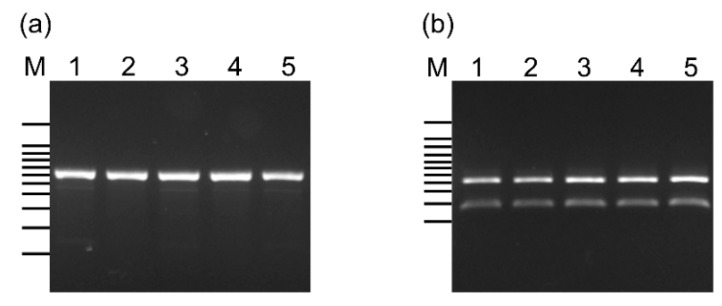
PCR amplification (**a**) and digestion with AseI (**b**) of the *Lactococcus lactis* subsp. *lactis gadB* gene. Lane M shows the 100-bp ladder marker from 100 to 1000 bp and 1500 bp and lanes 1–5 represent the CBW1, CBW2, CBW3, CBW4, and CBW5 strains, respectively.

The PCR amplicons of the CBW1–5 strains were approximately 600 bp in length (Figure 2a) with homologies to *L*. *lactis* subsp. *lactis* ATCC 19435 (GenBank accession number: AB067750) ranging from 97.6% to 98.8%. Their PCR products were digested by AseI endonuclease into two fragments of approximately 190 and 410 bp (Figure 2b).

### 3.3. Biochemical Characterization

The phenotypic characteristics of the strains are summarized in Table 5. The CBW1–5 isolates were identified as Gram-positive, non-motile, and catalase-negative bacterial strains that did not produce gas from glucose. The pH of Lactobacilli MRS broth decreased from 6.52 to 4.25–4.34 after two days of incubation. All five strains were grown on Lactobacilli MRS agar plates containing 1–7% sodium chloride at 37 °C for two days, but not on those containing more than 8% sodium chloride. Furthermore, all strains grew steadily in the broth at pH 4.0, 9.2, and 10.0, on the agar plate at 10 °C and at 40 °C, but not at 45 °C. All five strains utilized l-arabinose, ribose, d-xylose, galactose, d-glucose, d-fructose, d-mannose, mannitol, *N*-acetylglucosamine, amygdalin, arbutin, esculin, salicin, cellobiose, maltose, lactose, saccharose, trehalose, starch, *β*-gentiobiose, and gluconate (Table S2). 

### 3.4. Antimicrobial Activity Assay

The antimicrobial activity of the cell-free supernatant of Lactobacilli MRS broth (pH 7.0) obtained from five *L*. *lactis* subsp. *lactis* strains against the indicator bacterial species is shown in Table 2. Only the supernatant of strain CBW2 inhibited the growth of *V*. *alginolyticus* ATCC 17749 and *B*. *subtilis* subsp. *subtilis* ATCC 6051. The antimicrobial activity of strain CBW2 against *V*. *alginolyticus* and *B*. *subtilis* subsp. *subtilis* was completely eliminated by proteinase K treatment (*p* = 0.000001 and 0.000001, respectively), whereas it was only moderately diminished by treatment with trypsin (*p* = 0.000001 and 0.023102, respectively) and pepsin (*p* = 0.000001 and 0.024799, respectively) (Table 6). In contrast, lysozyme, catalase, and lipase treatments had no effect on antimicrobial activity (Table 6).

### 3.5. Genetic Evidence for Nisin Synthesis

**Figure 3:**
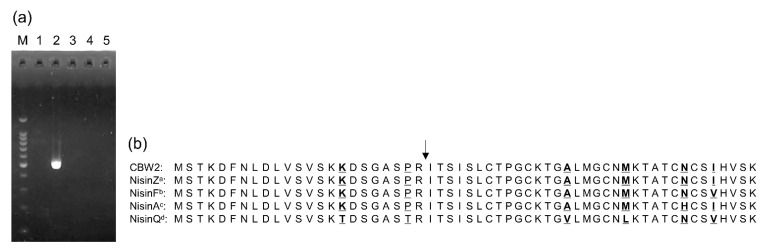
Specific PCR amplification of the structural gene for the nisin precursor (*nisA*) (**a**) and alignment of the proposed prepeptide sequences of the nisin variant (**b**). Lane M shows the 100-bp ladder marker from 100 to 1000 bp and 1500 bp and lanes 1–5 represent the CBW1, CBW2, CBW3, CBW4, and CBW5 strains, respectively. The National Center for Biotechnology Information (NCBI) GenBank accession numbers are: ^a^ CAA79467, ^b^ ABU45463, ^c^ CAA48380, and ^d^ BAG71479. Vertical arrow indicates a processing site. Substituted residues are marked in bold and underlined.

We obtained an amplicon only from strain CBW2, the size of which was similar to that found in a previous study [43], using the precursor nisin structural gene (*nisA*) primer set (Figure 3a). Fifty-seven amino acid residues (MSTKDFNLDLVSVSKKDSGASPRITSISLCTPGCKTGALMGCNMKTATCNCSIHVSK) in the DNA amplicon were deduced, and the predicted RiPP class was lanthipeptideA with a leader peptide (MSTKDFNLDLVSVSKKDSGASPR) and core peptide (ITSISLCTPGCKTGALMGCNMKTATCNCSIHVSK). The amino acid sequences of the nisin prepeptides highlighted that the deduced amino acid sequence from strain CBW2 was 100% coincident with that of nisin Z (Figure 3b). We also obtained amplicons only from strain CBW2 using each primer set targeting *nisB*, *nisC*, *nisT*, *nisP*, *nisF*, *nisI*, and *nisRK*, and their amplicons were the expected sizes based on a previous study [52] (Table 7, Figure S4).

## 4. Discussion

We successfully isolated *L*. *lactis* subsp. *lactis* from a fecal sample of a Cuvier’s beaked whale. This is the first study to reveal the characteristics of these whale-derived strains. We obtained *L*. *lactis*-specific PCR products from five strains (named CBW1–5), indicating that they are related to *L. lactis* [22,29]. Bacterial 16S rRNA gene analysis and *gadB* amplification with subsequent endonuclease digestion genetically identified strains CBW1–5 as *L*. *lactis* subsp. *lactis*. Their phenotypic properties, including the Gram reaction, motility, catalase activity, gas production from glucose, and carbohydrate fermentation profile, were consistent with those of marine-derived *L*. *lactis* subsp. *lactis* [22,53]. Similar to the characteristics of *L*. *lactis* subsp. *lactis* and unlike those of *L*. *lactis* subsp. *cremoris,* the strains could grow at pH 9.2, 40 °C, and in 4% sodium chloride [54]. Hence, the CBW1–5 isolates were identified as *L*. *lactis* subsp. *lactis*. In addition, the results of RAPD-PCR using the M13 primer indicated that the isolated strains were genotypically diverse and not clonal, despite their high 16S rRNA gene sequence similarities against the same bacteria species, i.e., *L*. *lactis* subsp. *lactis* JCM 5805^T^.

The transmission route of the identified *L*. *lactis* subsp. *lactis* strains to Cuvier’s beaked whale is intriguing. *L*. *lactis* strains have been isolated from various specimens, including plants [55], milk, and wastewater tanks [56]. In marine environments, a high-density colony of *L*. *lactis* was observed in the clone library of the intestine of grass puffer (*Takifugu niphobles*) [57], and halotolerant (6% sodium chloride) *L*. *lactis* subsp. *lactis* strains were isolated from coastal fish and clams (*Meretrix lamarckii*) [22,53]. These marine-derived strains may have diverged from terrestrial-derived strains through food chains and the water cycle [58]. The five *L*. *lactis* subsp. *lactis* strains isolated in this study showed higher halotolerance (up to 7% sodium chloride) than that of strains derived from plants, freshwater fish, and cheese [53,58], indicating their adaptation to the marine environment. In addition, the growth of all five strains at pH 4.0 and 10.0, as well as their carbohydrate fermentation profiles, was consistent with those of marine-derived strains [22,53]. However, they were different from those of freshwater fish-derived strains [21,58] in terms of their ability to ferment trehalose and inulin, and from the cheese-starter culture in their ability to ferment l-arabinose, mannitol, amygdalin, saccharose, and gluconate [22]. *L*. *lactis* subsp. *lactis* has been isolated from marine organisms, such as various fish species and Bivalvia [22,53], whereas Cuvier’s beaked whales found at Suruga Bay, Pacific coast in central Japan, mainly consume squids [59]. Thus, isolation of *L*. *lactis* subsp. *lactis* strains from squids and comparison of their genetic and phenotypic characteristics with those of Cuvier’s beaked whale-derived strains are required to reveal the transmission route of the bacteria.

Only the pH-neutralized, cell-free supernatant of Lactobacilli MRS broth (pH 7.0) obtained from the *L*. *lactis* subsp. *lactis* strain CBW2 inhibited the growth of *V*. *alginolyticus* ATCC 17749 and *B*. *subtilis* subsp. *subtilis* ATCC 6051, whereas its antimicrobial activity was abolished or diminished by treatment with proteinase K, trypsin, and pepsin, suggesting that its antimicrobial activity is not pH-related and may be associated with its peptidic nature. In contrast, no loss of activity was observed after treatment with lysozyme, lipase, and catalase, indicating that the inhibitory activity of strain CBW2 was not due to lipid, polysaccharide moieties, or hydrogen peroxide. In addition, strain CBW2 possesses the structural gene for the nisin precursor (*nisA*), and deduction of its sequence revealed 100% coincidence with that of nisin Z. These results suggest that strain CBW2 produces the precursor of nisin Z-like substances.

Nisin biosynthetic genes, which are located on a conjugative transposon, consist of 11 genes, including *nisA*, and are typically organized into four operons: *nisABTCIPRK*, *nisI*, *nisRK*, and *nisFEG* [60]. The genes are involved in nisin maturation (*nisBC*) [61], export from bacterial cells (*nisT*) [62], cleaving leader peptide (*nisP*) [63], self-induction of the *nisA* promotor by the mature nisin (*nisRK*) [64], anti-nisin protection (*nisI*) [65], and anti-nisin immunity function (*nisFEG*) [66]. Therefore, we examined each gene using previously reported primer sets. We found that strain CBW2 possessed nisin biosynthesis-related gene, *nisBTCIPRK*, and a nisin immunity-related gene, *nisF*, which is a part of the downstream *nisFEG* operon. Taken together, these data indicate that strain CBW2 produces the precursors of nisin Z-like substances, processes the precursors, then releases the mature nisins through exocytosis. In addition, the strain may protect itself from nisin and promote nisin production using the mature nisin product.

In general, nisin shows strong antibacterial effects against Gram-positive bacteria, but not against Gram-negative bacteria unless used in combination with physical treatment or substances, such as ethylenediaminetetraacetic acid, sodium citrate, and trisodium phosphate, to destabilize their outer membrane [9,12]. In contrast, *L*. *lactis* subsp. *lactis* A164 produces a nisin-like bacteriocin that inhibits *Salmonella typhimurium* (*S*. *typhimurium*) [67]. *L*. *lactis* WFLU12, which encodes the nisin Z gene cluster and colicin V [68], isolated from the olive flounder (*Paralichthys olivaceus*), showed antagonistic activity against *Edwardsiella tarda* (*E*. *tarda*) [24]. Kuwano et al. [13] reported that purified nisin Z could permeabilize both Gram-positive and Gram-negative bacterial cytoplasmic membranes, and that nisin’s antibacterial mechanism against Gram-negative bacteria may differ from that against Gram-positive bacteria. However, the detailed mechanism underlying nisin’s inhibition of Gram-negative bacteria has not been fully elucidated. In this study, the Cuvier’s beaked whale-derived *L*. *lactis* subsp. *lactis* strain CBW2 phenotypically and genetically showed the potential to synthesize nisin-like substances that may contribute to inhibiting the growth of *V*. *alginolyticus* and *B*. *subtilis* subsp. *subtilis*. The mechanism of *V*. *alginolyticus* growth inhibition, however, remains unclear. Purification and characterization studies of the nisin-like substance produced by strain CBW2 will provide useful information on the mechanism underlying nisin’s action against Gram-negative bacteria.

## 5. Conclusions

This is the first study to isolate and characterize *L. lactis* subsp. *lactis* strains from a fecal sample of a whale. The strains’ tolerance to NaCl and carbohydrate metabolism profiles suggest their adaptation to the marine environment and the whale’s conceivable acquirement of the strains through the food chain. The supernatant of strain CBW2 inhibited the growth of Gram-positive and -negative bacteria, but its inhibitory activity was significantly diminished by protease treatment. Furthermore, strain CBW2 possessed the structural gene for the nisin precursor (*nisA*), which was 100% identical to that for nisin Z, and nisin biosynthesis-related genes (*nisBTCIPRK* and *nisF*). These results suggest that strain CBW2 produces nisin-like substances; however, further studies are needed to purify and molecularly characterize the nisin-like substance from strain CBW2 to reveal the mechanism underlying the inhibition of harmful bacteria, particularly Gram-negative bacteria.

## Figures and Tables

**Table 1:** PCR primers used in this study.

Use	Primer Name	Gene	Sequence (5′ to 3′)	Reference
Whale species identification	t-Pro whale	mitochondrial DNA D-loop region	TCACCCAAAGCTGRARTTCTA	[27]
	Dlp5		CCATCGWGATGTCTTATTTAAGRGGAA	
*Lactococcus lactis*-specific PCR	8F	16S rRNA	AGAGTTTGATCCTGGCTCAG	[28]
	LacreR		GGGATCATCTTTGAGTGAT	[29]
Bacterial identification and phylogenetic analysis	27F	16S rRNA	AGAGTTTGATCMTGGCTCAG	[30]
	1492R		TACGGYTACCTTGTTACGACTT	
Classification of *Lactococcus lactis* subspecies	gadB21	*gadB*	CGTTATGGATTTGATGGATATAAAGC	[31]
	GAD7		ACTCTTCTTAAGAACAAGTTTAACAGC	

**Table 2:** Bacterial strains used for agar well diffusion assay and their sensitivity to the cell-free supernatant (pH 7.0) of five *Lactococcus lactis* subsp. *lactis* strains isolated from the fecal sample of a stranded female Cuvier’s beaked whale ^a^.

		Antimicrobial Activity of the Cell-Free Supernatant (pH 7.0)				
Indicator Bacteria Species	Strain	CBW1	CBW2	CBW3	CBW4	CBW5
*Vibrio alginolyticus*	ATCC 17749	−	++	−	−	−
*Vibrio parahaemolyticus*	ATCC 17802	−	−	−	−	−
*Escherichia coli*	DSM 30083	−	−	−	−	−
*Photobacterium damselae subsp. damselae*	DSM 7482	−	−	−	−	−
*Lactococcus lactis subsp. lactis*	ATCC 19435	−	−	−	−	−
*Lactococcus lactis subsp. cremoris*	ATCC 19257	−	−	−	−	−
*Lactococcus garvieae*	ATCC 43921	−	−	−	−	−
*Lactococcus plantarum*	ATCC 43199	−	−	−	−	−
*Lactococcus raffinolactis*	ATCC 43920	−	−	−	−	−
*Enterococcus faecalis*	DSM 20478	−	−	−	−	−
*Enterococcus hirae*	ATCC 8043	−	−	−	−	−
*Enterococcus faecium*	ATCC 19434	−	−	−	−	−
*Enterococcus canis*	DSM 17029	−	−	−	−	−
*Staphylococcus xylosus*	ATCC 29971	−	−	−	−	−
*Staphylococcus epidermidis*	ATCC 14990	−	−	−	−	−
*Bacillus subtilis subsp. subtilis*	ATCC 6051	−	+	−	−	−
*Streptococcus salivarius*	DSM 20560	−	−	−	−	−

^a^ −, no inhibition; +, inhibition zone diameter between 6 and 10 mm; ++, inhibition zone diameter exceeding 10 mm.

**Table 3:** PCR primers used to detect common bacteriocins produced by *L*. *lactis* strains and nisin biosynthesis-related genes.

Primer Name	Gene	Sequence (5′ to 3′)	Reference
Nis-F	*nisA*	CGGCTCTGATTAAATTCTGAAG	[43]
Nis-R		GGATTAGCTAGTAGTAACTGTTC	
Lact481-F	Lacticin 481	TCTGCACTCACTTCATTAGTTA	[44]
Lact482-R		AAGGTAATTACACCTCTTTTAT	
Lact3147-F	Lacticin 3147	TACTGGGGAAATAACGG	[45]
Lact3148-R		TGGACAAGTATTGGTAC	
Lcn972-F	Lactococcin 972	TTGTAGCTCCTGCAGAAGGAACATGG	[46]
Lcn973-R		GCCTTAGCTTTGAATTCTTACCAAAAG	
LactABM-F	Lactococcin A, B, and M	GAAGAGGCAATCAGTAGAG	[43,47]
LactA-R	Lactococcin A gene	GTGTTCTATTTATAGCTAATG	[43]
LactB-R	Lactococcin B gene	CCAGGATTTTCTTTGATTTACTTC	[43]
LactM-R	Lactococcin M gene	GTGTACTGGTCTAGCATAAG	[47]
p4	*nisB*	AGAGAAGTTATTTACGATCAAC	[48]
P5		ATCTGACAACAAATCTTTTTGT	
p6	*nisC*	TTCAGAGCAATATGAGG	[48]
p7		TATTAAGGCCACAATAAG	
P06-F	*nisT*	GAAGAATACATGAAATGAGG	[49]
P06-R		TAACTTTCCAGCTGTCCC	
P08-F	*nisI*	ATTGTGGCCTTAATAGGG	[49]
P08-R		TAGCGACTTGTCAGAAGC	
P11-F	*nisF*	CAGGTGCTACAAGATATCAG	[49]
P11-R		ACAACTCCGCAATACCATCAG	
Prim-NisP5	*nisP*	GGATTTGGTATCTGTTTCGAAG	[50]
Prim-NisP3		TCTTTCCCATTAACTTGTACTGTG	
Nis3	*nisRK*	CAGTGCCATGGGTAAAAAATATTCAATGCG	[51]
Nis4		CTTAGAGAATTCTCTAATGAG	

**Table 4:** Nearest phylogenetic neighbor of the five bacterial strains isolated from the fecal sample of a stranded Cuvier’s beaked whale according to the EzBioCloud 16S rRNA sequences database.

Strain	Sequence Length (bp)	Nearest Phylogenetic Neighbor	Similarity (%)	Completeness (%)
CBW1	1420	*Lactococcus lactis* subsp. *lactis* JCM 5805^T^	99.93	96.3
CBW2	1414		99.65	95.9
CBW3	1412		99.86	95.8
CBW4	1368		99.78	92.2
CBW5	1376		99.04	91.8

**Table 5:** Phenotypic characteristics of the five *Lactococcus lactis* subsp. *lactis* strains isolated from the fecal sample of a stranded female Cuvier’s beaked whale ^a^.

Characteristics	CBW1	CBW2	CBW3	CBW4	CBW5
Gram reaction	+	+	+	+	+
Motility	−	−	−	−	−
Catalase	−	−	−	−	−
Gas from glucose	−	−	−	−	−
Final pH ^b^	4.25	4.31	4.32	4.29	4.34
Growth					
NaCl (%)					
1	+	+	+	+	+
2	+	+	+	+	+
3	+	+	+	+	+
4	+	+	+	+	+
5	+	+	+	+	+
6	+	+	+	+	+
7	+	+	+	+	+
8	−	−	−	−	−
9	−	−	−	−	−
10	−	−	−	−	−
15	−	−	−	−	−
Temperature (°C)					
10	+	+	+	+	+
40	+	+	+	+	+
45	−	−	−	−	−
pH					
4.0	+	+	+	+	+
9.2	+	+	+	+	+
10.0	+	+	+	+	+

^a^ +, positive; −, negative. ^b^ Final pH of Lactobacilli MRS broth was measured after two days of incubation.

**Table 6:** Enzyme treatment effects on antimicrobial activity of the pH-neutralized, cell-free supernatant of strain CBW2 against *Vibrio alginolyticus* ATCC 17749 and *Bacillus subtilis* subsp. *subtilis* ATCC 6051.

	Diameter of the Inhibition Zone ± SD (mm)	
Enzyme	*Vibrio alginolyticus*	*Bacillus subtilis* subsp. *subtilis*
Control	15.13 ± 0.15	8.38 ± 0.34
Proteinase K	0.00 ± 0.00 **	0.00 ± 0.00 **
Trypsin	8.80 ± 0.20 **	7.86 ± 0.10 *
Pepsin	10.13 ± 0.12 **	7.87 ± 0.25 *
Lysozyme	15.13 ± 0.31 ^N.S.^	8.23 ± 0.16 ^N.S.^
Catalase	14.97 ± 0.25 ^N.S.^	8.30 ± 0.20 ^N.S.^
Lipase	14.83 ± 0.15 ^N.S.^	8.26 ± 0.25 ^N.S.^

* and ** represent statistically significant differences compared with the control at *p* < 0.05 and < 0.01, respectively. ^N.S.^ indicates no significant differences.

**Table 7:** PCR results for nisin biosynthesis-related genes in five *Lactococcus lactis* subsp. *lactis* strains isolated from the fecal sample of a stranded female Cuvier’s beaked whale ^a^.

Gene	CBW1	CBW2	CBW3	CBW4	CBW5
*nisA*	−	+	−	−	−
*nisB*	−	+	−	−	−
*nisC*	−	+	−	−	−
*nisT*	−	+	−	−	−
*nisI*	−	+	−	−	−
*nisF*	−	+	−	−	−
*nisP*	−	+	−	−	−
*nisRK*	−	+	−	−	−

^a^ +, positive; −, negative.

## Data Availability

The 16S rRNA sequences of the five strains obtained in this study are registered in the DDBJ/GenBank/EMBL databases under the accession numbers LC570855–LC570859.

## Supplementary Materials

The following are available online at https://www.mdpi.com/2076-2607/9/2/243/s1. Table S1: Bacterial strains and the culture temperature and conditions used for the agar well diffusion assay. Table S2: Carbohydrate utilization profiles of the five strains detected in this study and those of reference strains determined using API 50 CH. Figure S1: The sampled stranded female Cuvier’s beaked whale (a) and the location where it was found in Shimizu, Shizuoka, Japan (b). Figure S2: PCR amplification using a universal primer (8F, [28]) and a *Lactococcus lactis*-specific primer (LacreR, [29]). Lane M: 100-bp ladder marker from 100 to 1000 bp and 1500 bp; lanes 1–5: strains CBW1–5. Figure S3: RAPD-PCR bands patterns of five *Lactococcus lactis* subsp. *lactis* strains. Lane M: molecular weight ladder marker; lanes 1–5: strains CBW1–5. Figure S4: PCR results for nisin biosynthesis-related genes in five *Lactococcus lactis* subsp. *lactis* strains isolated from the fecal sample of a stranded female Cuvier’s beaked whale. Lane M: 100-bp ladder marker from 100 to 1000 bp and 1500 bp; lanes 1–5: strains CBW1–5.
