# Supplementary material for: Antimicrobial Activity of Lactococcus lactis subsp. lactis Isolated from a Stranded Cuvier’s Beaked Whale (Ziphius cavirostris) against Gram-Positive and -Negative Bacteria
Source: Microorganisms. 2021 Jan 25;9(2):243. doi: 10.3390/microorganisms9020243 (PMC7911499; doi:10.3390/microorganisms9020243)
Supplement: Supplementary file 1 [file microorganisms-09-00243-s001.pdf]

Table S1. Bacterial strains and the culture temperature and conditions used for the agar well diffusion assay.

Indicator bacteria species	Strain	Culture temperature	Culture condition
Vibrio alginolyticus	ATCC 17749	25°C	aerobic
Vibrio parahaemolyticus	ATCC 17802	25°C	aerobic
Escherichia coli	DSM 30083	25°C	aerobic
Photobacterium damselae subsp. *damselae*	DSM 7482	25°C	aerobic
Lactococcus lactis subsp. *lactis*	ATCC 19435	25°C	anaerobic
Lactococcus lactis subsp. *cremoris*	ATCC 19257	30°C	anaerobic
Lactococcus garvieae	ATCC 43921	25°C	anaerobic
Lactococcus plantarum	ATCC 43199	30°C	anaerobic
Lactococcus raffinolactis	ATCC 43920	30°C	anaerobic
Enterococcus faecalis	DSM 20478	30°C	anaerobic
Enterococcus hirae	ATCC8043	30°C	anaerobic
Enterococcus faecium	ATCC 19434	30°C	anaerobic
Enterococcus canis	DSM 17029	37°C	anaerobic
Staphylococcus xylosus	ATCC 29971	37°C	anaerobic
Staphylococcus epidermidis	ATCC 14990	37°C	anaerobic
Bacillus subtilis subsp. *subtilis*	ATCC 6051	37°C	aerobic
Streptococcus salivarius	DSM 20560	37°C	anaerobic

Table S2. Carbohydrate utilization profiles of the five strains detected in this study and those of reference strains determined using API 50 CH.

Sugar assay	CBW1	CBW2	CBW3	CBW4	CBW5	Marine fish-isolates[a]	Bivalvia-isolates[bc]	Freshwater fish-isolates[d]
2-keto-gluconate	−	−	−	−	−	−	−	−
5-keto-gluconate	−	−	−	−	−	−	−	−
Adonitol	−	−	−	−	−	−	−	−
Amygdalin	+	+	+	+	+	+	+	+
Arbutin	+	+	+	+	+	+	+	+
Cellobiose	+	+	+	+	+	+	+	+
D-Arabinose	−	−	−	−	−	−	−	−
D-Arabitol	−	−	−	−	−	−	−	−
D-Fructose	+	+	+	+	+	+	+	+
D-Fucose	−	−	−	−	−	−	−	−
D-Glucose	+	+	+	+	+	+	+	+
D-Lyxose	−	−	−	−	−	−	−	−
D-Mannose	+	+	+	+	+	+	+	+
D-Raffinose	−	−	−	−	−	−	−	−
D-Tagatose	−	−	−	−	−	−	8%	−
D-Turanose	−	−	−	−	−	−	−	−
D-Xylose	+	+	+	+	+	+	75%	−
Dulcitol	−	−	−	−	−	−	−	−
Erythritol	−	−	−	−	−	−	−	−
Esculin	+	+	+	+	+	+	+	+
Galactose	+	+	+	+	+	+	+	+
Gluconate	+	+	+	+	+	+	92%	+
Glycerol	−	−	−	−	−	−	−	−
Glycogen	−	−	−	−	−	−	−	−
Inositol	−	−	−	−	−	−	−	−
Inulin	−	−	−	−	−	−	−	+
L-Arabinose	+	+	+	+	+	+	33%	+
L-Arabitol	−	−	−	−	−	−	−	−
L-Fucose	−	−	−	−	−	−	−	−
L-Sorbose	−	−	−	−	−	−	−	−
Lactose	+	+	+	+	+	+	+	+
L-Xylose	−	−	−	−	−	−	−	+
Maltose	+	+	+	+	+	+	+	+
Mannitol	+	+	+	+	+	+	42%	+
Melezitose	−	−	−	−	−	−	−	−
Melibiose	−	−	−	−	−	−	−	−
N-acetylglucosamine	+	+	+	+	+	+	+	+

Rhamnose	−	−	−	−	−	−	8%	−
Ribose	+	+	+	+	+	+	+	+
Saccharose	+	+	+	+	+	+	92%	+
Salicin	+	+	+	+	+	+	+	+
Sorbitol	−	−	−	−	−	−	−	−
Starch	+	+	+	W	W	+	58%	+
Trehalose	+	+	+	+	+	+	+	−
Xylitol	−	−	−	−	−	−	−	−
α-Methyl-D-glucoside	−	−	−	−	−	−	−	−
α-Methyl-D-mannoside	−	−	−	−	−	−	−	−
β-Gentiobiose	+	+	+	+	+	+	+	+
β-Methyl-xyloside	−	−	−	−	−	−	−	−

+; positive reaction, W; weakly positive reaction, and -; negative reaction

[a] The data for coastal fish-derived *Lactococcus lactis* subsp. *lactis* was cited from the result of Itoi *et al*. [22]

[b] The data for clam (*Meretrix lamarckii*)-derived *Lactococcus lactis* subsp. *lactis* was cited from the result of Itoi *et al*. [53]

[c] The percentage values represent the positive incidence

[d] The data for freshwater fish-derived *Lactococcus lactis* subsp. *lactis* was isolated from Amur catfish (*Silurus asotus*) [21], and the data is shown in Itoi *et al*. [58]

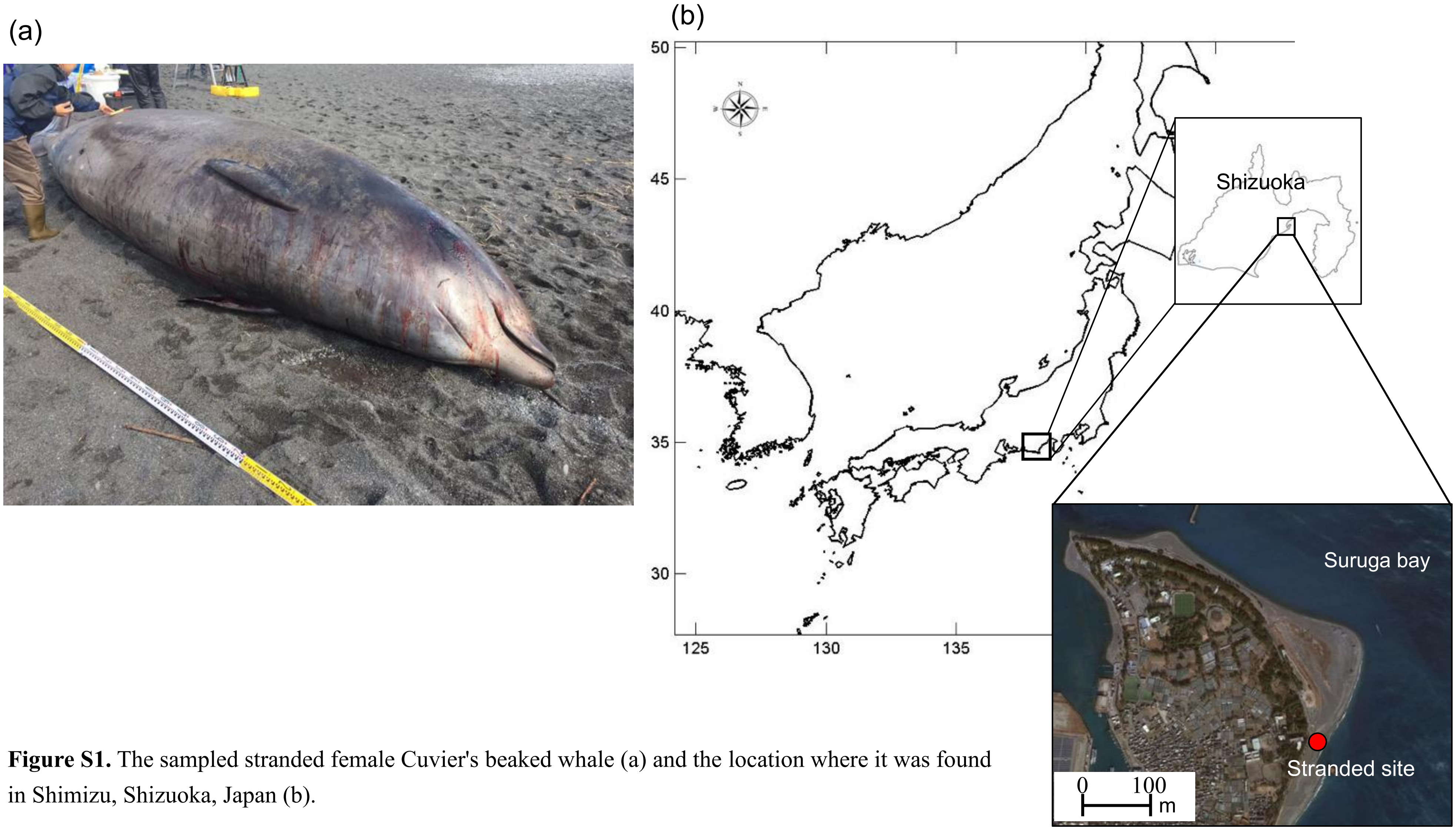

Figure S1. The sampled stranded female Cuvier's beaked whale (a) and the location where it was found in Shimizu, Shizuoka, Japan (b).

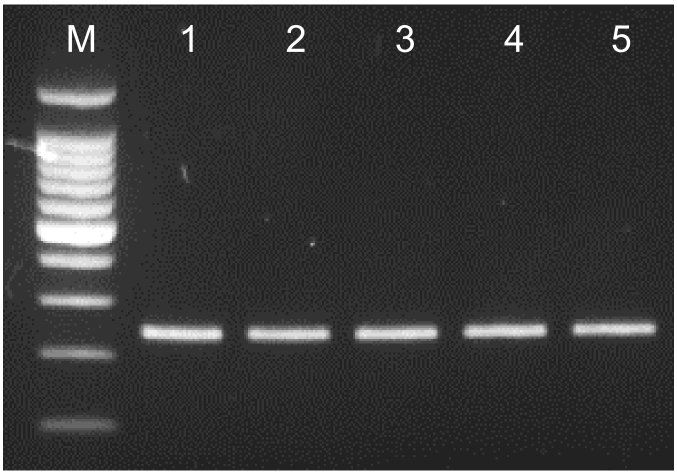

Figure S2. PCR amplification using a universal primer (8F, [28]) and a *Lactococcus lactis*-specific primer (LacreR, [29]). Lane M: 100 bp ladder marker from 100 to 1,000 bp and 1,500 bp; lanes 1–5: strains CBW1–5.

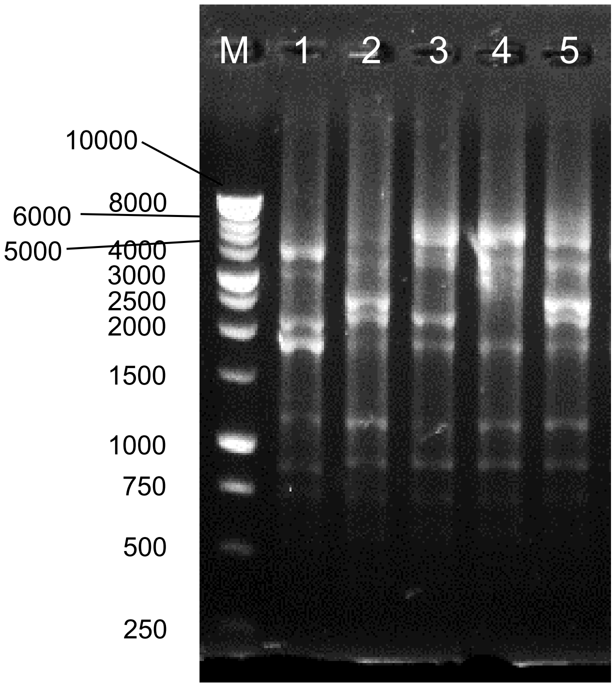

Figure S3. RAPD-PCR bands patterns of five *Lactococcus lactis* subsp. *lactis* strains in this study. Lane M: molecular weight ladder marker; lanes 1-5: strains CBW1-5.

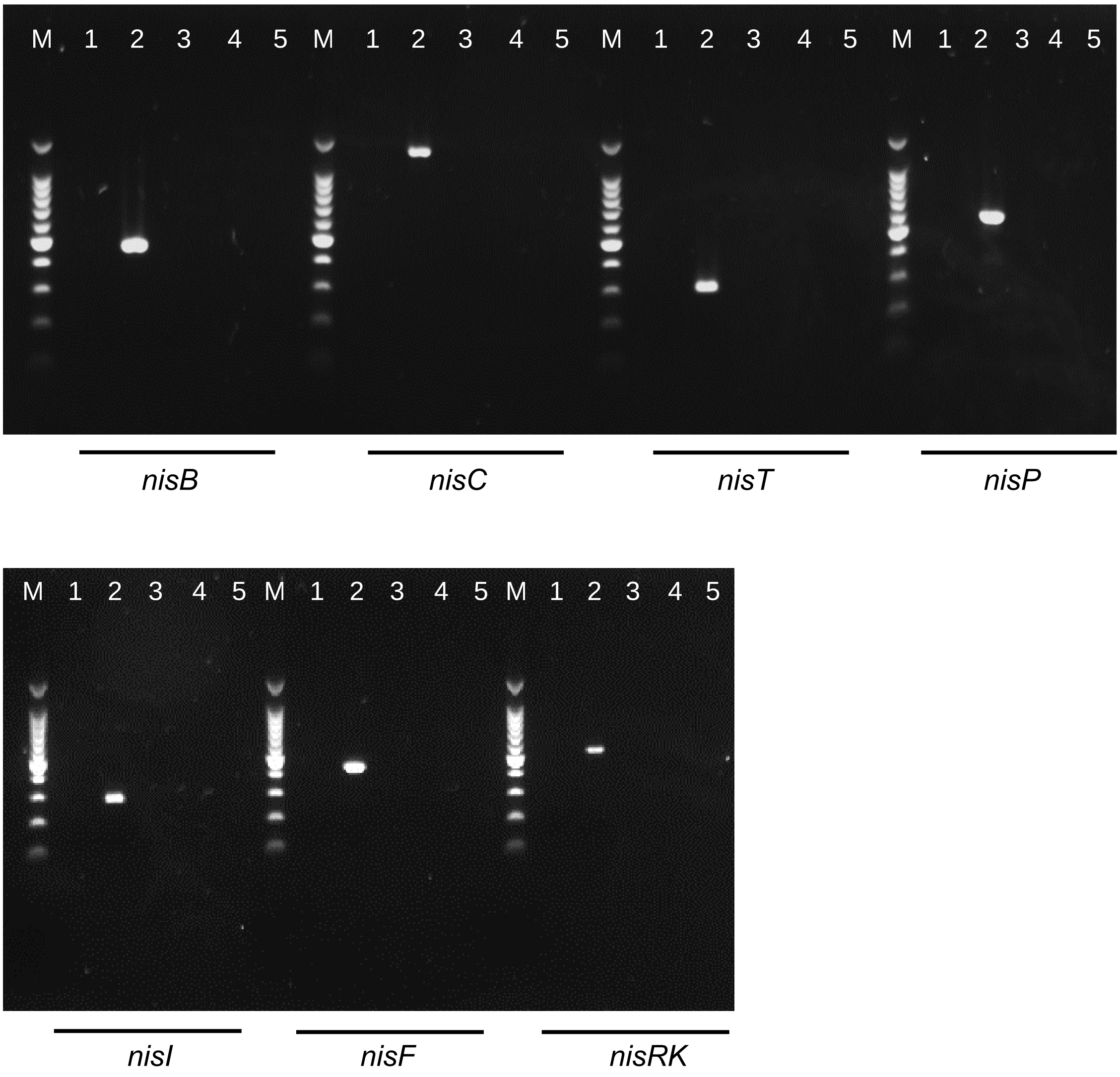

Figure S4. PCR results for nisin biosynthesis-related genes in five *Lactococcus lactis* subsp. *lactis* strains isolated from the fecal sample of a stranded female Cuvier's beaked whale.Lane M: 100-bp ladder marker from 100 to 1,000 bp and 1,500 bp; lanes 1-5: strains CBW1-5.
